# Characteristics of the gut microbiome in esports players compared with those in physical education students and professional athletes

**DOI:** 10.3389/fnut.2022.1092846

**Published:** 2023-01-16

**Authors:** Maria Kulecka, Barbara Fraczek, Aneta Balabas, Paweł Czarnowski, Natalia Zeber-Lubecka, Barbara Zapala, Katarzyna Baginska, Maria Glowienka, Monika Szot, Maciek Skorko, Anna Kluska, Magdalena Piatkowska, Michał Mikula, Jerzy Ostrowski

**Affiliations:** ^1^Department of Gastroenterology, Hepatology and Clinical Oncology, Centre of Postgraduate Medical Education, Warsaw, Poland; ^2^Department of Genetics, Maria Sklodowska-Curie National Research Institute of Oncology, Warsaw, Poland; ^3^Department of Sports Medicine and Human Nutrition, Institute of Biomedical Sciences, University of Physical Education, Krakow, Poland; ^4^Department of Biochemistry, Radioimmunology and Experimental Medicine, The Children's Memorial Health Institute, Warsaw, Poland; ^5^Department of Clinical Biochemistry, Faculty of Medicine, Jagiellonian University Medical College, Krakow, Poland; ^6^Department of Sports Dietetics, Gdansk University of Physical Education and Sport, Gdansk, Poland; ^7^Institute of Psychology, Polish Academy of Sciences, Warsaw, Poland

**Keywords:** shotgun metagenomics, metabolomics, esport players, professional athletes, diet, physical activity

## Abstract

**Introduction:**

Esports is a category of competitive video games that, in many aspects, may be similar to traditional sports; however, the gut microbiota composition of players has not been yet studied.

**Materials and methods:**

Here, we investigated the composition and function of the gut microbiota, as well as short chain fatty acids (SCFAs), and amino acids, in a group of 109 well-characterized Polish male esports players. The results were compared with two reference groups: 25 endurance athletes and 36 healthy students of physical education. DNA and metabolites isolated from fecal samples were analyzed using shotgun metagenomic sequencing and mass spectrometry, respectively. Physical activity and nutritional measures were evaluated by questionnaire.

**Results:**

Although anthropometric, physical activity and nutritional measures differentiated esports players from students, there were no differences in bacterial diversity, the Bacteroidetes/Firmicutes ratio, the composition of enterotype clusters, metagenome functional content, or SCFA concentrations. However, there were significant differences between esports players and students with respect to nine bacterial species and nine amino acids. By contrast, all of the above-mentioned measures differentiated professional athletes from esports players and students, with 45 bacteria differentiating professional athletes from the former and 31 from the latter. The only species differentiating all three experimental groups was *Parabacteroides distasonis*, showing the lowest and highest abundance in esports players and athletes, respectively.

**Conclusion:**

Our study confirms the marked impact of intense exercise training on gut microbial structure and function. Differences in lifestyle and dietary habits between esports players and physical education students appear to not have a major effect on the gut microbiota.

## 1. Introduction

The colonic microbiota in healthy adults is dominated by the phyla Firmicutes and Bacteroidetes (which comprise over 90% of the bacteria), and to a lesser extent by Actinobacteria, Proteobacteria, Verrucomicrobia, and Fusobacteria. The gut phyla comprises at least 1,800 different genera, and 15,000–36,000 bacterial species, which contain ~10 million non-redundant microbial genes (1, 2). This complex ecosystem trains the immune system, protects against opportunistic pathogens, harvests nutrients and energy from the diet, and degrades dietary carbohydrates, lipids, and proteins, thereby producing metabolites with both local and systemic actions (3). The relative abundance of gut bacterial strains is impacted by host genotype and ethnicity, age, and sex, and is highly associated with changes in environmental factors such as diet, physical activity, sanitation, illness, and therapies (4–7). It is not surprising, therefore, that changes in the functional composition of the gut microbiota and its metabolites are associated with several disorders, including obesity-related disorders (3, 6–8). The microbiome of obese individuals is less rich than that of normal body weight individuals, the latter being characterized by changes in the relative abundance of the two dominant phyla: Bacteroidetes and Firmicutes (4, 9). In turn, physical exercise correlates with increased richness and diversity of the gut microbiota, a higher abundance of health-promoting bacteria such as *Faecalibacterium prausnitzii, Roseburia hominis, Akkermansia muciniphila*, and *Prevotella* species, and an increase in beneficial microbial-associated metabolites, mostly short chain fatty acids (SCFAs) (10–13).

According to the Central Statistical Office, a Polish system of official statistics (http://www.stat.gov.pl/), the estimated number of video games players in Poland in 2021 reached 16 million, and about 2.6 million are esports enthusiasts; the number of sports clubs that have esports sections in their structures or create programs to support esports is growing. It is difficult to estimate the exact number of gamers worldwide, as the gaming industry is constantly evolving. However, according to a report by Newzoo, the global gaming market is expected to generate $184.4 billion in 2022 with a forecast of growth to $211.2 billion by 2025 (14). Esports, like traditional sports, require a combination of physical and mental skills. However, esports players seem to rely more on cognitive abilities to achieve success (15, 16). Searching for excellence, esports players from universities in the United States practice between 5.5 and 10 h a day before competitions; however, 40% do not participate in any kind of physical activity (17). Moreover, prolonged gaming and associated physical inactivity can lead to malnutrition or overeating, and inappropriate eating habits (18, 19). Male collegiate esports players are significantly less active, and have a higher body-fat percentage, with lower lean body mass and bone mineral content, than non-esports players (20). Other negative consequences of esports gaming include upper limb dysfunction, neck, back, or wrists pain, vision problems, metabolic dysregulation, and sympathetic overstimulation (21–23). However, evidence on whether esports players are more or less physically active than the general population is controversial, as esports players may include physical training as a part of their training regimen and to manage stress (24); indeed, 92.7% of 260 male professional esports players with the normal body mass index (BMI) reported high or moderate levels of physical activity (21). The top 10% of higher-ranked esports players were more physically active than the bottom 90% of players (25); however, 80.3% of 1,772 esports players representing five esports across all skill levels were not meeting the WHO physical activity guidelines at the same rates as others in the general population.

To identify objective measures of factors related to the lifestyle, physical activity, and dietary habits of esports players, we used shotgun metagenomic sequencing and small molecule mass spectrometry (MS) to investigate the gut microbiota and bacterial metabolites, as well as SCFAs and amino acids, in a group of well-characterized Polish male esports players. The results were compared with those obtained from two reference groups: endurance athletes and healthy students of physical education. This is the first metagenomic study exploring esports players' gut microbiome.

## 2. Materials and methods

### 2.1. Participants

This study was conducted in accordance with the principles of the Declaration of Helsinki and was approved by Maria Sklodowska-Curie National Research Institute of Oncology Local Bioethics Board (decision 54/2017). All participants provided informed consent to participate. Between July 2020 and July 2021, 109 male esports players were recruited at the peak of their gaming season. In addition, 36 male education physical education students were recruited as a control group (University of Physical Education, Cracow) and 25 professional athletes (eight marathon runners, three cross-country skiers, and 14 football players) were assigned to an independent endurance sports player group. Of these, 59 and 36 esports players and students, respectively, completed dietary questionnaires.

The inclusion criteria were the professional level of esportsmanship (for esports players), self-declaration of a good health condition and remaining on a Western-type diet without specific dietary restrictions (for esports players and students). Due to the dominance of men in esports, women were excluded from the study. None of the participants received antibiotic treatment in the last 6 months before entering the study.

### 2.2. Collection of data regarding physical activity and nutritional habits

#### 2.2.1. Evaluation of physical activity

By definition, the physical activity of professional athletes was extremely high. The Polish version of the International Physical Activity Questionnaire was used to measure the physical activity of esports players and students of physical education (26). The questions in the questionnaire referred to the time the subjects spent sitting, walking, or performing moderate to vigorous physical activity for at least 10 min (without interruption) over the last 7 days. Activities that lasted <10 min at a time were not considered. Energy expenditure (expressed as MET-min/week) was calculated by multiplying the number of days of activity per week by the corresponding MET value and the average duration of that activity (in minutes). The results for all activities undertaken during the week were then summed to obtain the weekly energy expenditure. Based on the results, participants were assigned to one of three physical activity levels: (1) insufficient (low), physical activity that did not meet the conditions for sufficient or high levels; (2) sufficient, when individuals met one of the following criteria: three or more days of intense physical exertion of not <20 min per day, or five or more days of moderate exertion or walking for not <30 min per day, or five or more days of any combination of physical activity (walking, moderate or intense exertion) exceeding 600 MET-min/week; and (3) high, when individuals meet one of the following criteria: three or more days of intense exercise, totaling at least 1,500 MET-min/week, or seven days of any combination of exertion (walking, moderate, or intense exertion) exceeding 3,000 MET-min/week.

#### 2.2.2. Evaluation of the energy and nutritional value of the diet

A quantitative dietary assessment was performed *via* a thee day running diary method and a weighing method. Participants recorded a food diary listing all foods and beverages consumed for 3 consecutive days, or on 3 days selected at random, specifying their size in grams or home measures (using a detailed table of home measures and grams of foods and selected foods). Before monitoring, all subjects were trained to ensure correct dietary recording, which consisted of specifying precisely the type of meal consumed (breakfast, second breakfast, lunch, afternoon tea, dinner), the time of consumption, and the food products and dishes (including the method of preparation).

The Remote Food Photography Method was used if there were difficulties estimating the portion size consumed. This involved taking digital photographs of food before and after consumption and then sending them to the study author to estimate the amount of a portion consumed (compared with a standard serving size) (27). The completed food diaries were analyzed for accuracy and correctness, and participants were asked to fill in any gaps. All dietary data were then entered into the 'Aliant. Dietary Calculator' (version 2.0) assesses the energy value and nutrient content of foods. The average intake of macronutrients (protein, fat, total carbohydrates), fatty acids, fiber, and cholesterol in both groups was related to normal values for the Polish population (28), and recommendations for athletes (29–32), as well as the average supply of selected micronutrients, were related to normal values for the level of safe (Estimated Average Requirement, EAR), recommended daily amounts, and adequate intake (AI) (28).

### 2.3. Metagenomics and metabolomics procedures

Fecal samples were collected from all participants. The stool was self-collected using a stool specimen collection kit consisting of a Styrofoam box containing a sterile tube with a spatula and an ice pack. Following collection, the samples were transported at −20°C to the laboratory and stored at −80°C until use.

#### 2.3.1. Metagenomics

DNA was isolated using the QIAamp Fast DNA Stool Mini Kit protocol (Qiagen, Hilden, Germany), as described previously (33). The quality and quantity of the extracted DNA was assessed by detecting the optical density using a NanoDrop 2000/2000c Spectrophotometer (Thermo Fisher Scientific, Carlsbad, CA, USA) and a fluorometric-based method using the Qubit dsDNA HS Assay Kit (Thermo Fisher Scientific, Carlsbad, CA, USA), respectively. DNA libraries were prepared and sequenced at the CeGaT GmbH genomic service (Tübingen, Germany) on the Illumina NovaSeq 6,000 Platform using 100 bps paired-end reads.

#### 2.3.2. Short-chain fatty acid and amino acid profiling

Metabolites were extracted and derivatized as described (34). Briefly, 100 mg of reach frozen stool sample was placed in a 2 ml tube containing ceramic beads and 1 ml of 10% iso-butanol. The sample was mechanically homogenized and centrifuged, and 675 μl of supernatant was transferred to a new Eppendorf tube. After adding 125 μl of 20 mM NaOH and 400 μl chloroform to the sample, it was vortexed and centrifuged. A 400 μl aliquot of the upper aqueous phase was transferred to a new tube, followed by the addition of 100 μl of pyridine and 80 μl of isobutanol. The volume of each sample was adjusted to 650 μl with ultra-pure water. Calibration standards of SCFAa (formate, acetate, propionate, butyrate, isobutyrate, and valerate) and amino acids (alanine, L-arginine, L-cystine, L-glutamic acid, L-leucine, L-lysine, L-serine, L-threonine, L-tyrosine, L-valine, and L-histidine) were obtained from Sigma-Aldrich (St. Louis, MO). Samples and standards were derivatized with chloroformate isobutyl (50 μl per 650 μl sample or standard). The samples were vortexed and after adding 170 μl, hexane were vortexed again and centrifuged. An aliquot of the upper isobutyl-hexane phase was transferred to an autosampler vial. Gas chromatographic analysis of the fecal extracts was performed on an Agilent 7000D Triple Quadrupole mass spectrometer coupled to a 7,890 GC System with a G4513A autosampler (Agilent Technologies, Santa Clara, CA, USA). A VF-5ms column (30 m, 0.25 mm, 0.50 μm) was used for analysis. The temperatures of the injector, ion source, quadrupole, and transfer line were set to 260, 250, 150, and 275°C, respectively. The carrier gas was helium and the flow rate was 1 mL/min. The derivatized sample was injected into the VF-5ms column with a split ratio of 50:1, and the solvent delay was set to 3 min. The oven temperature program started at 40°C, where it was kept for 5 min before increasing to 275°C at a rate of 10°C/min, where it was held for 10 min. The total run time was 38.5 min. MS data were gathered in full scan mode from m/z 15–650 at a frequency of 4.9 scans per second. MassHunter software (Agilent Technologies, Santa Clara, CA, USA) was used for analysis.

### 2.4. Statistical analysis

Shannon diversity indices were calculated using the diversity function in the vegan package (version 2.5-7) (35). Values were compared using the Kruskal-Wallis or Mann-Whitney U-test (for two groups only). Bacterial taxa were assigned with Metaphlan3 (36), version 3.0.13, with default parameters. Enterotypes were assigned according to the methods described by Arumugam et al. (37), using the R code present at https://enterotype.embl.de/enterotypes.html. Enterotype drivers were assigned based on NetConfer (38) analysis of correlation coefficient weighted networks. Fisher's exact test verified the relationship between the experimental group and enterotype. *Post-hoc* analysis was performed according to the methods described by Shan and Gerstenberger (39). Differences in taxa abundance between groups were assessed using the LINDA [Linear (Lin) Model for Differential Abundance (DA)] (40) method for compositional data, with *p*-values corrected using the Benjamini–Hochberg (41) procedure to minimize the false discovery rate (FDR) and using relative abundance values, computed by Metaphlan3. The Bacteroidetes/Firmicutes ratios were compared using the Kruskal-Wallis or Mann-Whitney U-tests (for two groups only). Differences in metabolite concentrations between study groups and enterotypes were assessed using the Kruskal-Wallis test (with an FDR-corrected Mann-Whitney U-test as a *post-hoc* test).

Functional assignments were conducted by human version 3.0 (part of BioBakery Workflows) (36), using MetaCyc (42) pathways as a reference database. Quality filtering and decontamination were performed with KneadData as a part of the functional analysis. The LINDA method was used to assess compositional data, with *p-*values corrected by the Benjamini–Hochberg procedure to minimize the FDR.

## 3. Results

Altogether, the study included three groups of participants: 109 esports players and two reference groups (36 physical education students and 25 professional athletes).

Sixty-three esports players answered the questionnaire regarding gaming experience. They trained and participated in League of Legends (LOL) (46%), Counter Strike: Global Offensive (CS:GO) (52.4%), and Starcraft (1.6%); 13 and 87% reported having professional (receiving financial gratification and training under the supervision of a trainer) and semi-professional experience, respectively. Professionals spent more time playing than semi-professionals (6 ± 1.95 h/day *vs*. 4 ± 1.15 h/day, respectively); the gaming experience of professional players averaged 6.4 ± 2.6 years. Mechanics, tactics, communication, and movement precision were the main contents of the training sessions, which took place daily or several times a week. The weekly training session of CS:GO players consisted of watching two video replays (demos) and participating in 20 practice matches (approximately 3–4 h in total), while official games were played in BO3 mode (about 1–1.5 h). League of Legends players participated in three-team training sessions (scrims) per day (lasting approximately 4–6 h) and took part in Solo Q, a 1 vs. 1 ranking game against randomly assigned non-team players. Esports games are played on various platforms, i.e., consoles, computers, and mobile phones, and using different control devices (computer mouses, keyboards, and gamepads), either live in front of an audience or *via* an online broadcast. Among esports players, 60.3 and 17.3% were university students and secondary school students, respectively (Supplementary Table 1).

As summarized in Supplementary Table 2, the average age of esports players was significantly lower than that of students (21 *vs*. 23 years, *p* < 0.001). The body mass, height, and BMI did not differ between esports players and students. However, esports players demonstrated significantly lower muscle mass (*p* < 0.001) and higher fat mass (*p* < 0.001) than students. While the self-reported physical activity of esports players, estimated as energy expenditure, was significantly lower (*p* < 0.01), their self-reported physical activity level was higher (*p* < 0.05) than that of students.

### 3.1. Dietary variables

Dietary variables of esports players and students were converted to nutrient intake values (Supplementary Table 3). The energy value of the esports players' diet was significantly lower (*p* < 0.01) than that of the students' diet (2,063 kcal *vs*. 2,418 kcal, respectively). Evaluation of energy balance showed poor balance (in terms of energy intake) in the majority of esports players (70.49%) and students (66.67%). The total protein content and the amount of plant and animal protein in the regular diet of esports players was significantly lower than in the student group. This is a mean (from 3 consecutive days) daily intake (as mentioned in the methodology section) (gg). Total carbohydrate intake was also significantly lower (*p* < 0.001) for esports players than for students. There was no statistically significant difference in the intake of fat and trans-fatty acids in both groups, although the diet of esports players had a lower fat content than that of students). Evaluation of the average intake of macronutrients, expressed in grams per kilogram of body weight (g/kg bw), showed that the average protein intake of esports players was significantly lower (*p* < 0.05) than that of students. Intake of total carbohydrates, fructose, and sugars was also significantly lower in esports players than in students; 8.1 *vs*. 10.4%, respectively), although there was no difference in dietary fat content). Nutrient standards of sugars consumption are expressed as a percentage of energy intake. The supply of saturated fatty acids was higher in esports players (12.01 *vs*. 9.97%) than in students, and polyunsaturated fatty acids (PUFAs) and cholesterol levels were lower (2.97 *vs*. 3.92%,). As above, nutrient standards are expressed as a percentage of energy intake. Esports players consumed a significantly lower amount of fiber than the student group. It was noted that both esports players and students showed a tendency to avoid alcohol (consumption was expressed in grams and grams per kilogram bw).

### 3.2. Evaluation of the gut microbiome, SCFAs, and amino acids in esports players, students, and professional athletes

**Figure 1:**
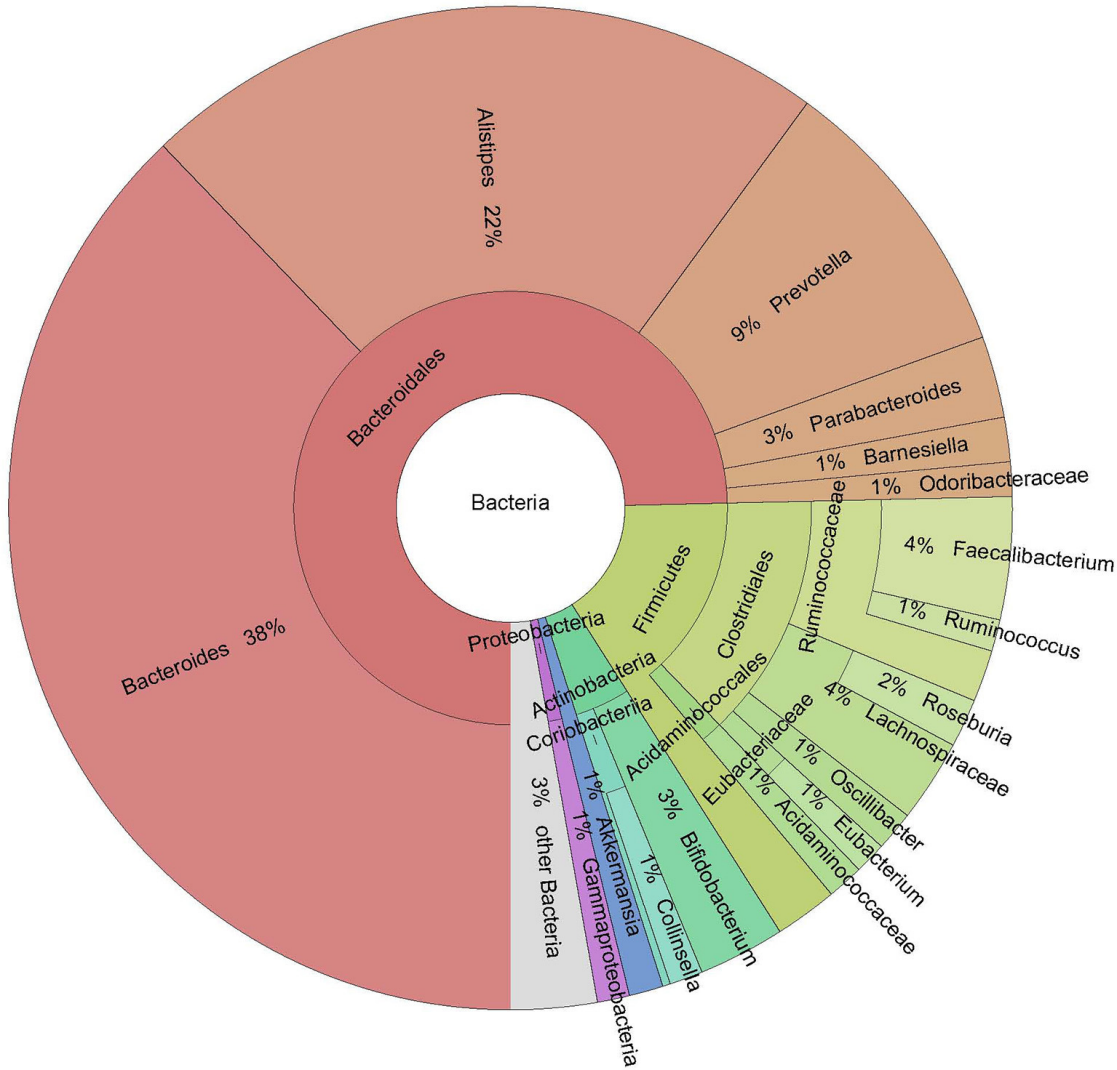
Krona plot of taxa with a mean abundance higher than 1%.

On average, 43 million reads were generated per sample (median = 43 million). On average, five out of 13 detected phyla (Actinobacteria, Bacteroidetes, Firmicutes, Proteobacteria, and Verrucomicrobia) comprised more than 1% of the microbiome. The dominant phylum was Bacteroidetes, with a mean abundance of 75.45%. Of the 136 genera detected, 13 had a mean abundance higher than 1%; of these, the five most prevalent were *Bacteroides, Alistipes, Prevotella, Faecalibacterium*, and *Bifidobacterium* (Figure 1). On the species level, 382 species were detected.

### 3.3. Enterotype-related changes

**Figure 2:**
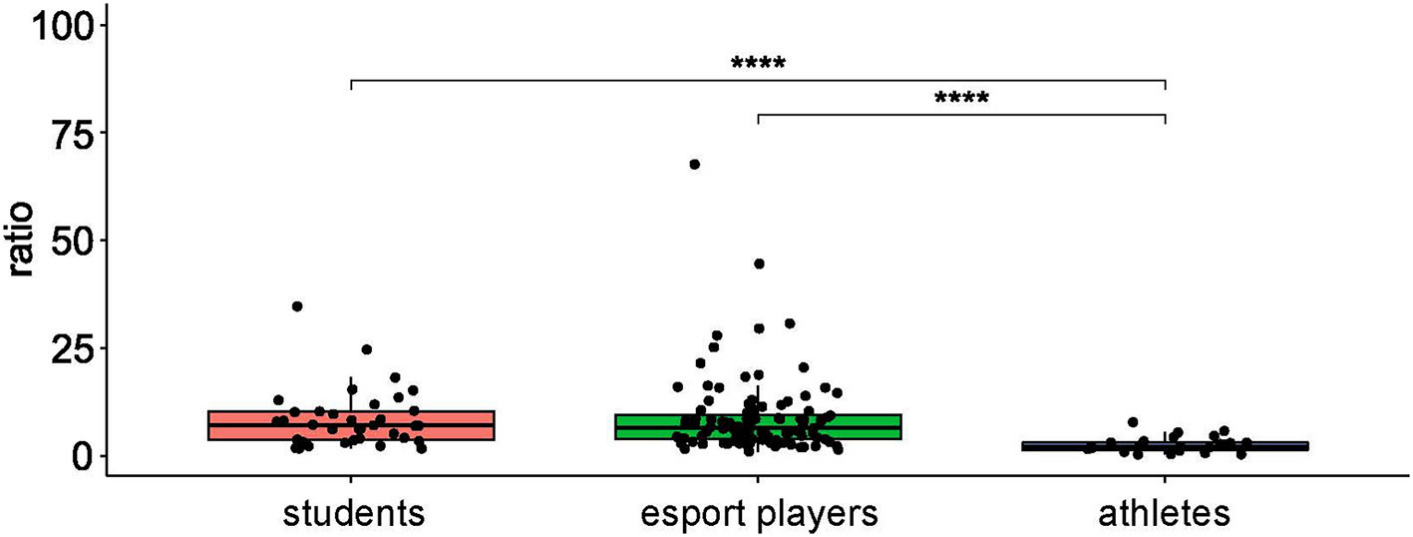
The Bacteroidetes:Firmicutes ratio in healthy controls, professional athletes, and esportsmen. ****: *p* ≤ 0.0001.

SparCC correlation analysis using NetConfer identified three enterotypes, of which enterotype 1 was *Bacteroides*-driven and enterotype 3 was *Prevotella*-driven. While both of those taxa had the most significant degrees and betweenness levels in their respective enterotype networks, enterotype 2 shared similar degrees of *Alistipes, Bacteroides*, and *Bifidobacterium* in the network (Supplementary Figure 1, Supplementary Table 4). As presented in Table 1, while the enterotype frequency was almost identical in esports players and students, enterotype 1 was not found in professional athletes. In contrast, enterotypes 2 and 3 were significantly more prevalent in athletes than in esports players and students (Table 1). Furthermore, professional athletes exhibited a significantly lower B/F ratio than both esports players and students (Figure 2).

**Table 1:** Enterotype abundance in students, esports players, and professional athletes.

	**1 (B)**	**2 (A)**	**3 (P)**
Students	16 (44%)	13 (36%)	7 (19%)
Esports players	48 (44%)	40 (37%)	20 (18%)
Profesional athletes	0^***^	15 (60%)^*^	10 (40%)^*^

* p < 0.05,

^**^p < 0.01,

*** p < 0.001.

When esports players and students were subdivided according to physical activity level, we found statistically significant differences in enterotype distribution (*p* = 0.0005, Fisher's exact test with a simulated *p*-value). *Bacteroides*-associated enterotype 1 was over-represented and *Alistipes*-associated enterotype 2 was under-represented among participants with the lowest levels of activity when compared with those with a medium activity level (Table 2).

**Table 2:** Enterotype distribution in different physical activity levels in combined groups of esports players and students.

**Physical activity**	**Enterotype**		
	**1 (B)**	**2 (A)**	**3 (P)**
Level 1	34 (52%)^**^	18 (28%)^**^	13 (20%)
Level 2	15 (38%)	21 (53%)	4 (10%)^*^
Level 3	8 (35%)	8 (35%)	7 (30%)

* p < 0.05,

** p < 0.01,

^***^p < 0.001; level 1 – low; level 2 – medium; level 3 – high physical activity.

### 3.4. Bacterial diversity

**Figure 3:**
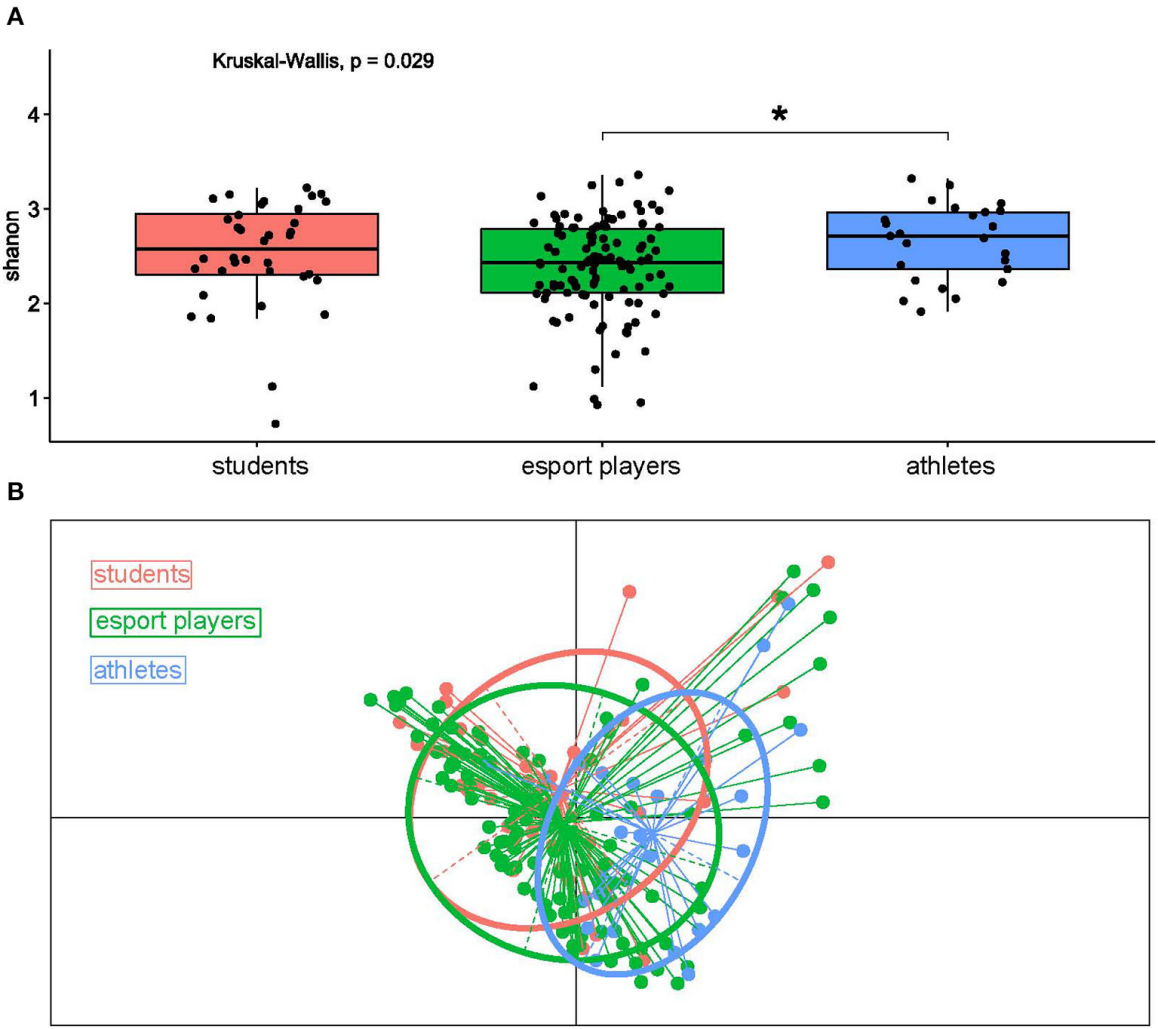
**(A)** α-diversity, as measured by the Shannon index, in students, esports players, and professional athletes. **(B)** PCoA, based on Bray Curtis distances, of students, esports players, and professional athletes. *: p ≤ 0.05.

After correction by multiple hypothesis testing, neither α- nor β-diversity differed esports players from students, while the α-diversity differed esports players from professional athletes (*p* = 0.052, Mann-Whitney U-test, Figure 3A), and there were statistically significant community dissimilarities between both esports players and students, and professional athletes, as measured by ANOSIM (Analysis of Similarities) (R = 0.14, *p* = 0.0013, Figure 3B, Table 3).

**Table 3:** Community dissimilarities, as measured by ANOSIM R (number of permutations: 9999).

**Comparison**	**R**	**Significance**
Esport players *vs*. students	−0.00546	5.25E-01
Esport playars *vs*. professional athletes	0.2719	1.00E-04
Students *vs*. professional athletes	0.2288	7.00E-04

### 3.5. Bacterial abundance

**Figure 4:**
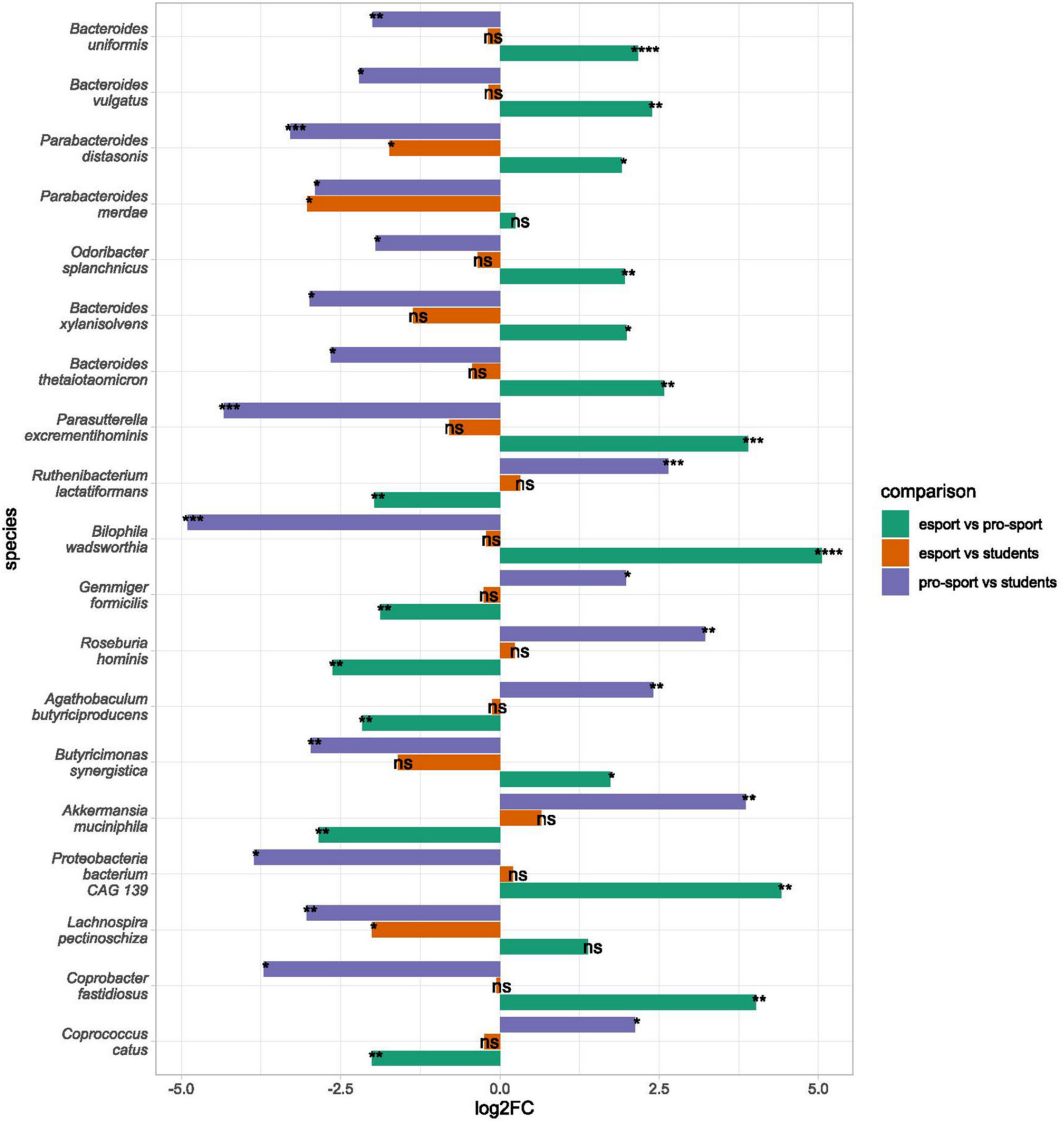
Differential taxa in bacterial species in esports players, professional athletes, and students. Bars indicate log2 from fold change (by the LINDA model. Significant changes are marked with asterisks as follows: ). Only species differential in at least two comparisons are presented. Species are sorted according to their mean abundance. ns: *p* > 0.05, *: *p* ≤ 0.05, **: *p* ≤ 0.01, ***: *p* ≤ 0.001, ****: *p* ≤ 0.0001.

On the species level, nine bacteria (*Parabacteroides distasonis, Lachnospira pectinoschiza, Parabacteroides merdae, Bacteroides faecis, Paraprevotella xylaniphila, Veillonella atypica, Veillonella parvula, Streptococcus salivarius*, and *Streptococcus parasanguinis*) differentiated esports players from students, and 31 and 45 bacteria differentiated professional athletes from students and esports players, respectively. *Parabacteroides distasonis* was the only bacteria that differentiated all groups; professional athletes had a lower abundance than esports players and students, while esports players had a lower abundance than students (Figure 4, Supplementary Table 5). Species belonging to the genera *Parabacteroides* and *Bacteroides* were under-represented in professional athletes. Esports players were differentiated from professional athletes mainly by the abundance of bacteria belonging to the *Bacteroides* genus, which was over-represented in esports players.

### 3.6. SCFA levels

**Figure 5:**
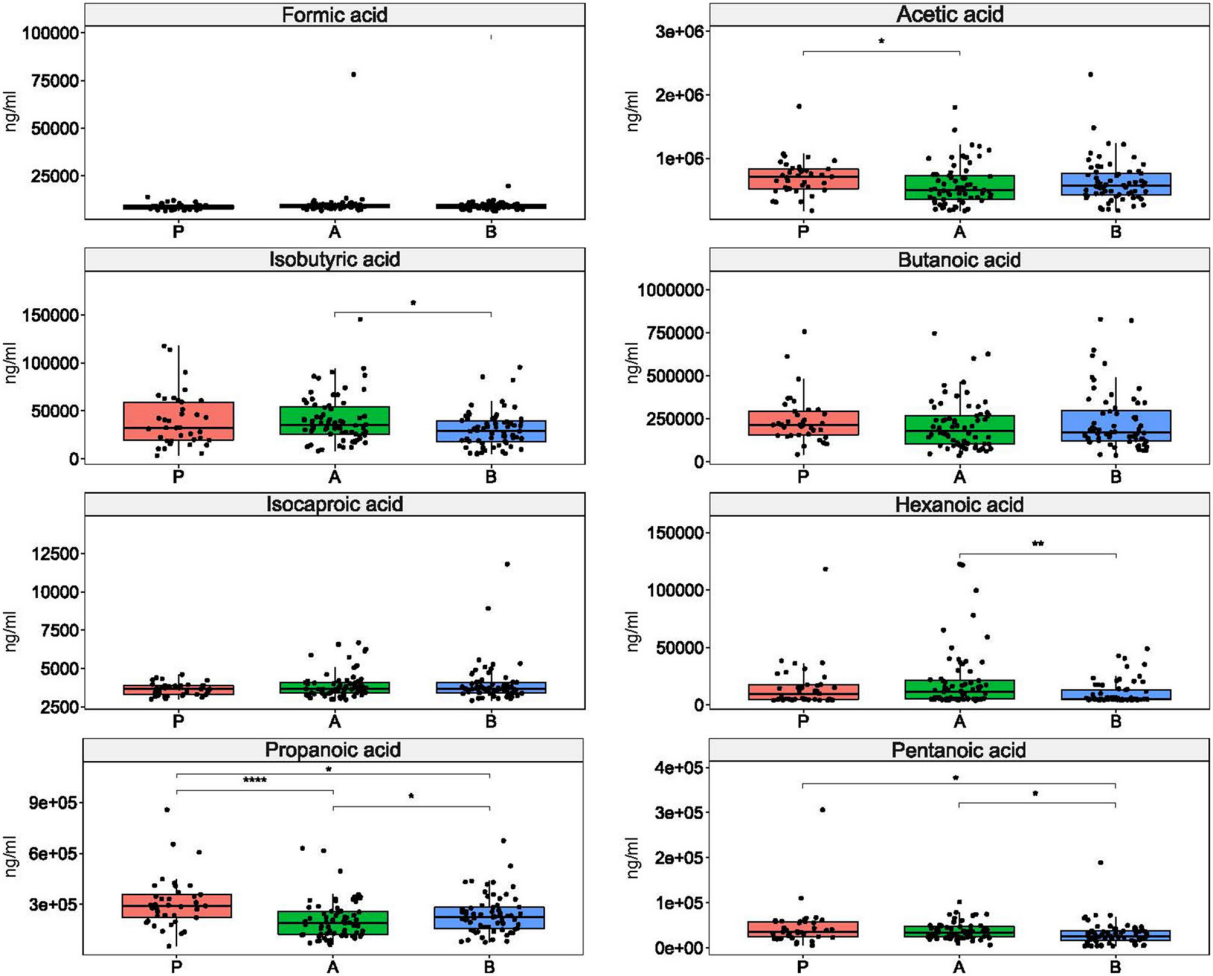
Differences in short chain fatty acid concentrations between enterotypes. *: *p* ≤ 0.05, **: *p* ≤ 0.01, ****: *p* ≤ 0.0001.

While the concentration of non-SCFAs differentiated the study groups (Supplementary Table 6), five SCFAs differentiated the enterotypes of esports players and students subdivided according to physical activity levels. Of these, isobutyric, pentanoic, and hexanoic acids differentiated between enterotypes 1 and 2; acetic acid differentiated between enterotypes 2 and 3; and pentanoic acid differentiated between enterotypes 1 and 3. Propanoic acid differentiated all enterotypes (Figure 5).

### 3.7. Amino acid levels

**Figure 6:**
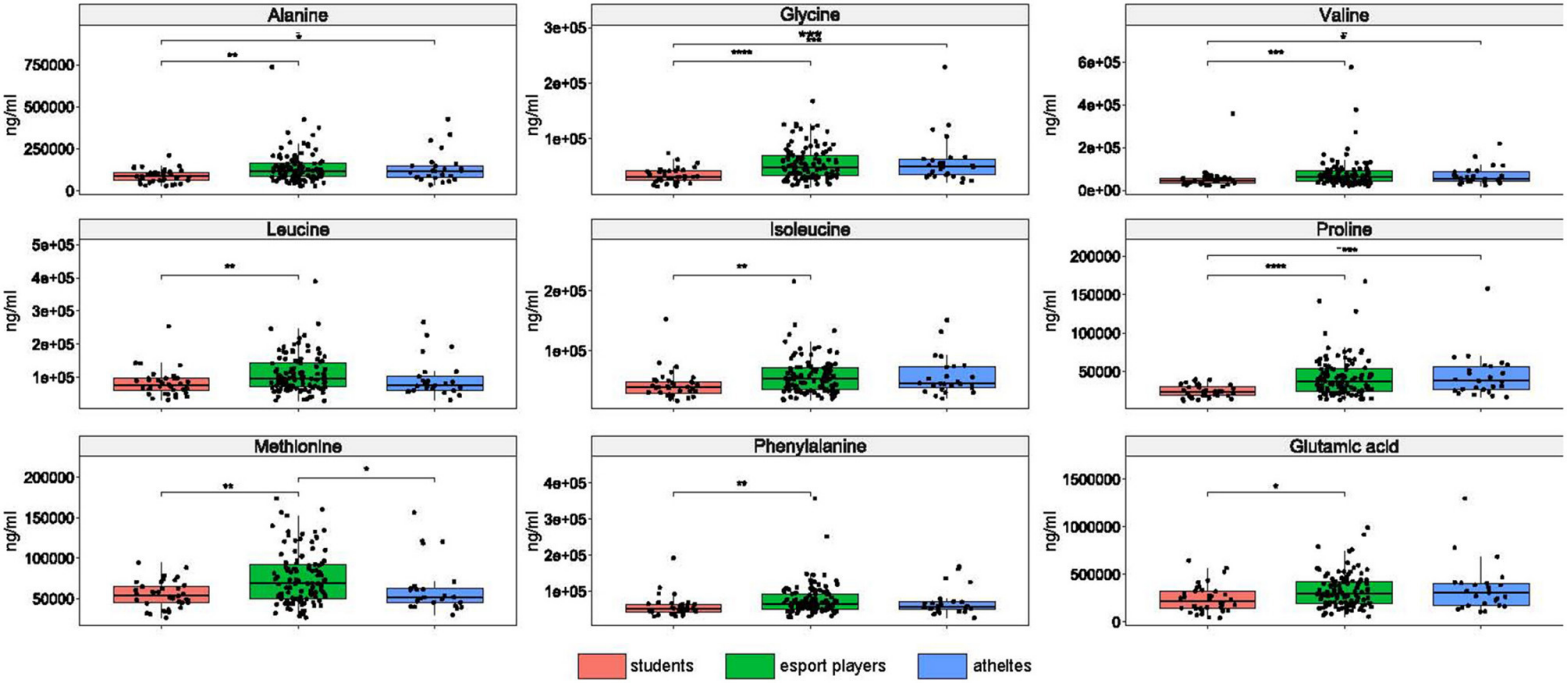
Differences in amino acid concentrations between the groups. *: *p* ≤ 0.05, **: *p* ≤ 0.01, ***: *p* ≤ 0.001, ****: *p* ≤ 0.0001.

The concentration of each of nine amino acids differentiated esports players from students, four amino acids differentiated professional athletics from students, and methionine differentiated esports players from both students and athletes (Figure 6).

**Figure 7:**
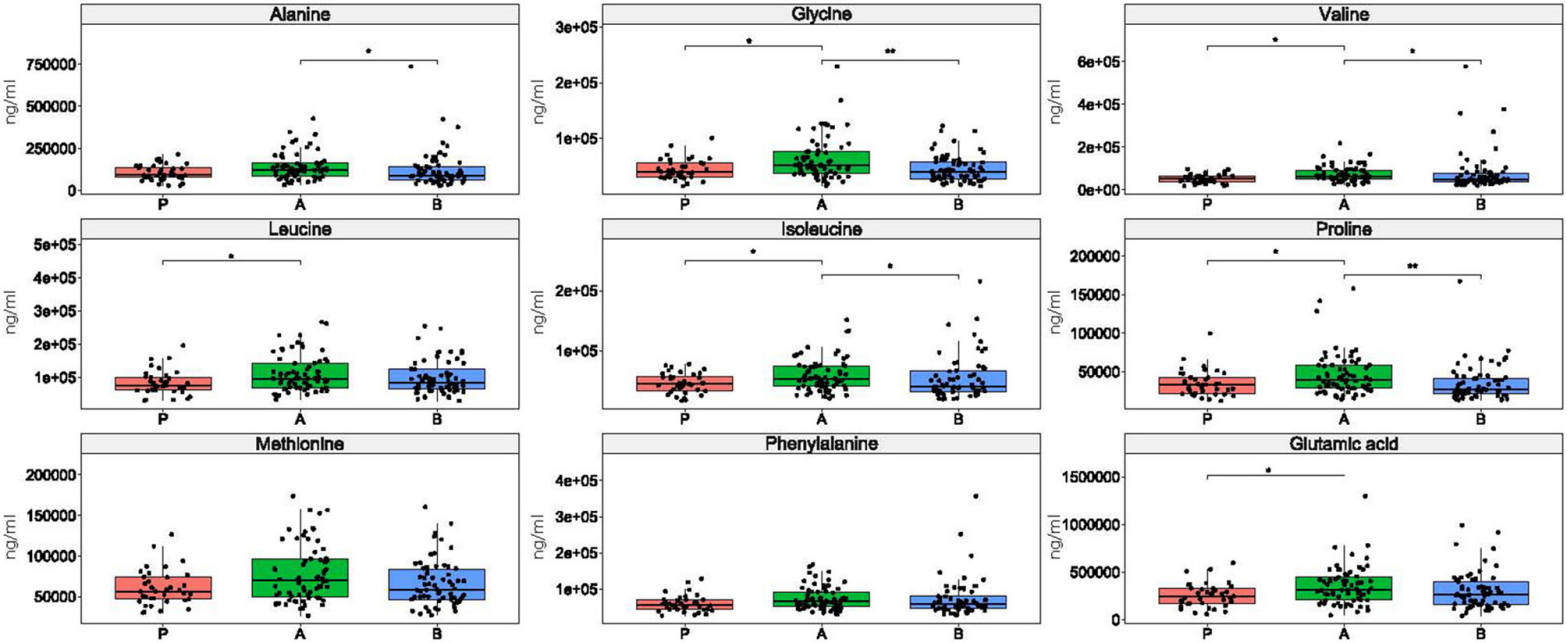
Differences in amino acid concentration between enterotypes. *: *p* ≤ 0.05, **: *p* ≤ 0.01.

Only phenylalanine did not differentiate between enterotypes in esports players and students (Figure 7). The Kruskal-Wallis test identified methionine as being statistically significant, but neither pairwise comparison was significant, probably due to loss of statistical power. Alanine differentiated enterotypes 1 and 2, leucine and glutamic acid differentiated enterotypes 2 and 3, and glycine, valine, isoleucine, and proline differentiated enterotype 2 from the remaining two enterotypes (Figure 7).

### 3.8. Correlation analysis between bacteria and metabolites

**Figure 8:**
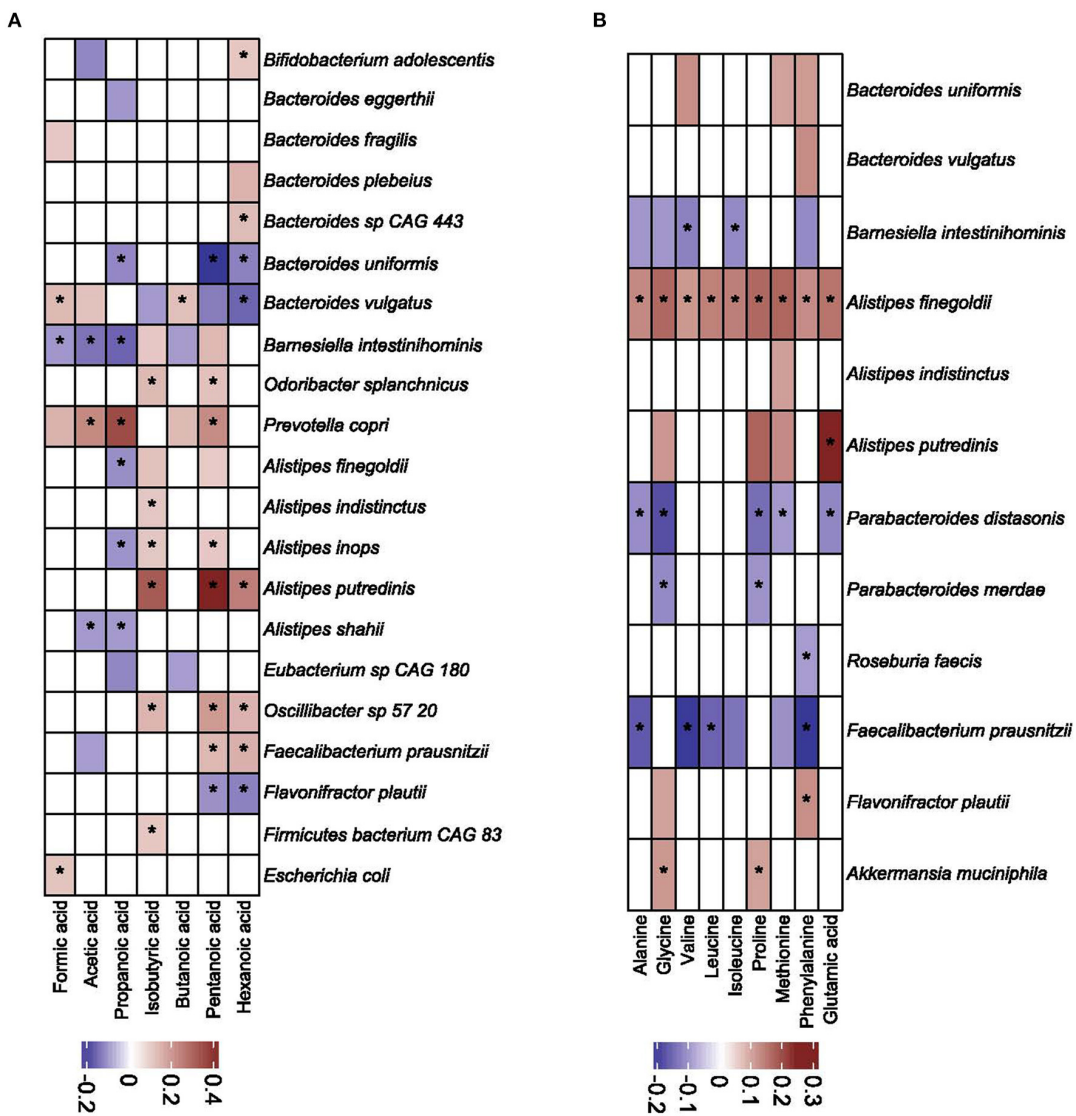
Correlation coefficients (as calculated by the sparCC algorithm) between bacterial taxa and short chain fatty acids **(A)** and amino acids **(B)**. Only values higher than 0.1 are shown. Values with corresponding *p-*value lower than 0.05 are denoted with asterisk.

We calculated the sparCC correlation coefficient to evaluate the correlation between the abundance of bacteria and the concentrations of SCFAs and amino acids. Of 161 bacterial species with a mean abundance > 0.01%, 21 (including genus *Bacteroides*, and *Prevotella*) (Figure 8A) and 12 species (Figure 8B) correlated with SCFAs and amino acids, respectively. *Bacteroides vulgaris, Barnesiella intestinihominis*, and *Prevotalla copri* correlated with at least five of seven SCFAs studied, and *Alistipes finegoldi* correlated positively with all nine amino acids studied. *Faecalibacterium prausnitzi* correlated negatively with seven amino acids.

### 3.9. Microbiome-related functional analyses

**Figure 9:**
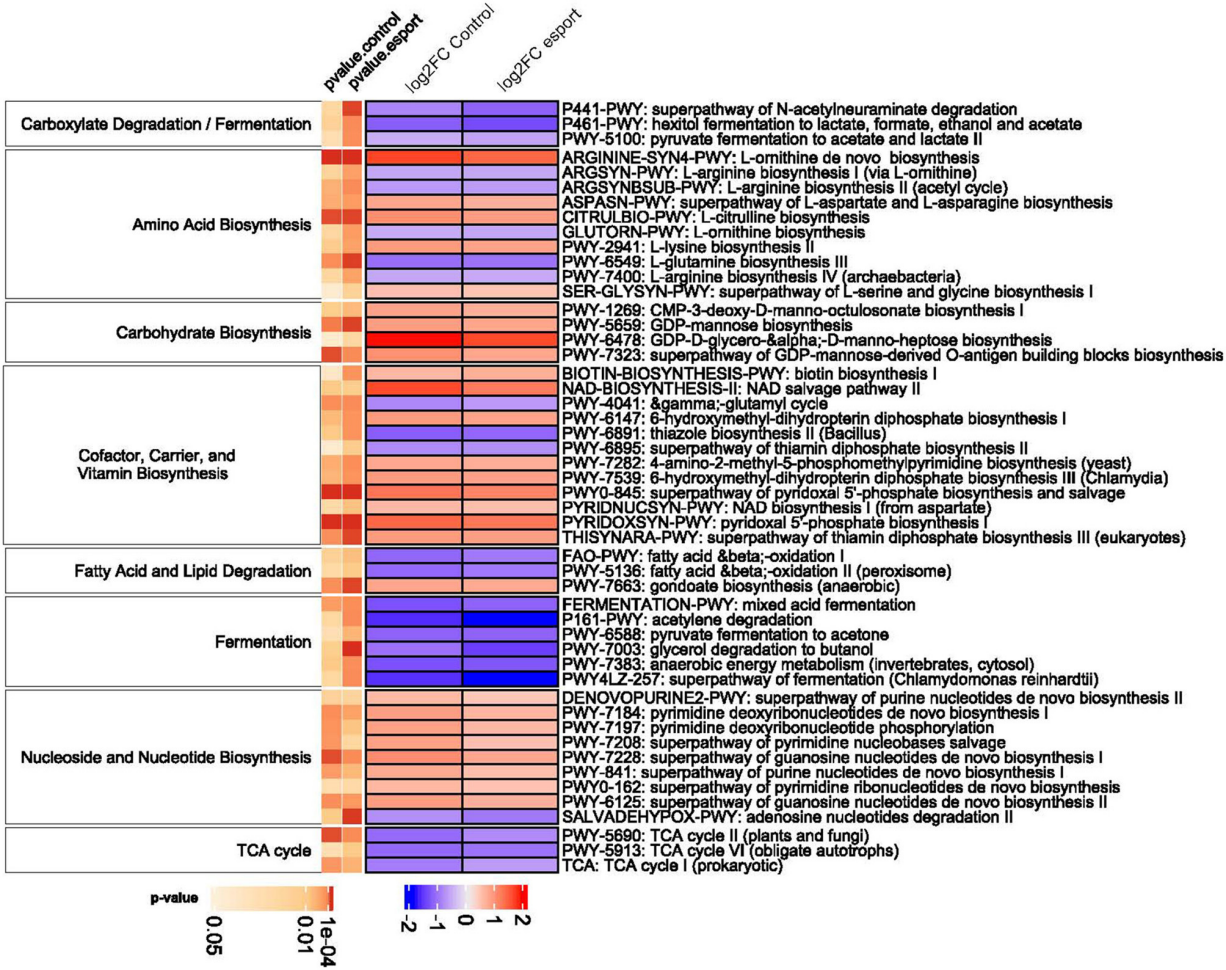
Pathways differentiating between the three groups (grouped by their super pathways). The heatmap demonstrates log2-transformed data. Only categories with at least three pathways are shown. P-.

Metagenome functional content was assessed using the MetaCyc pathway database. In total, 370 pathways were tested to make functional comparisons between esports players, students, and professional athletes. While we found no functional differences between esports players and students, 77 and 158 metabolic pathways differentiated professional athletes from students and esports players, respectively (Supplementary Table 7, Figure 9). Of these, 71 pathways were common in both comparisons. The differential pathways mainly were associated with *Fermentation, Amino acid Biosynthesis and Degradation, Carbohydrate Biosynthesis and Degradation, Fatty Acid Biosynthesis and Degradation*, and *TCA Cycle* pathways. Of note, compared with athletes, all differential pathways associated with *Fermentation*, the *TCA Cycle*, and *Fatty Acid beta-Oxidation* were under-represented in students and esports players.

### 3.10. Correlations between dietary variables, gut taxa, and metabolites

**Figure 10:**
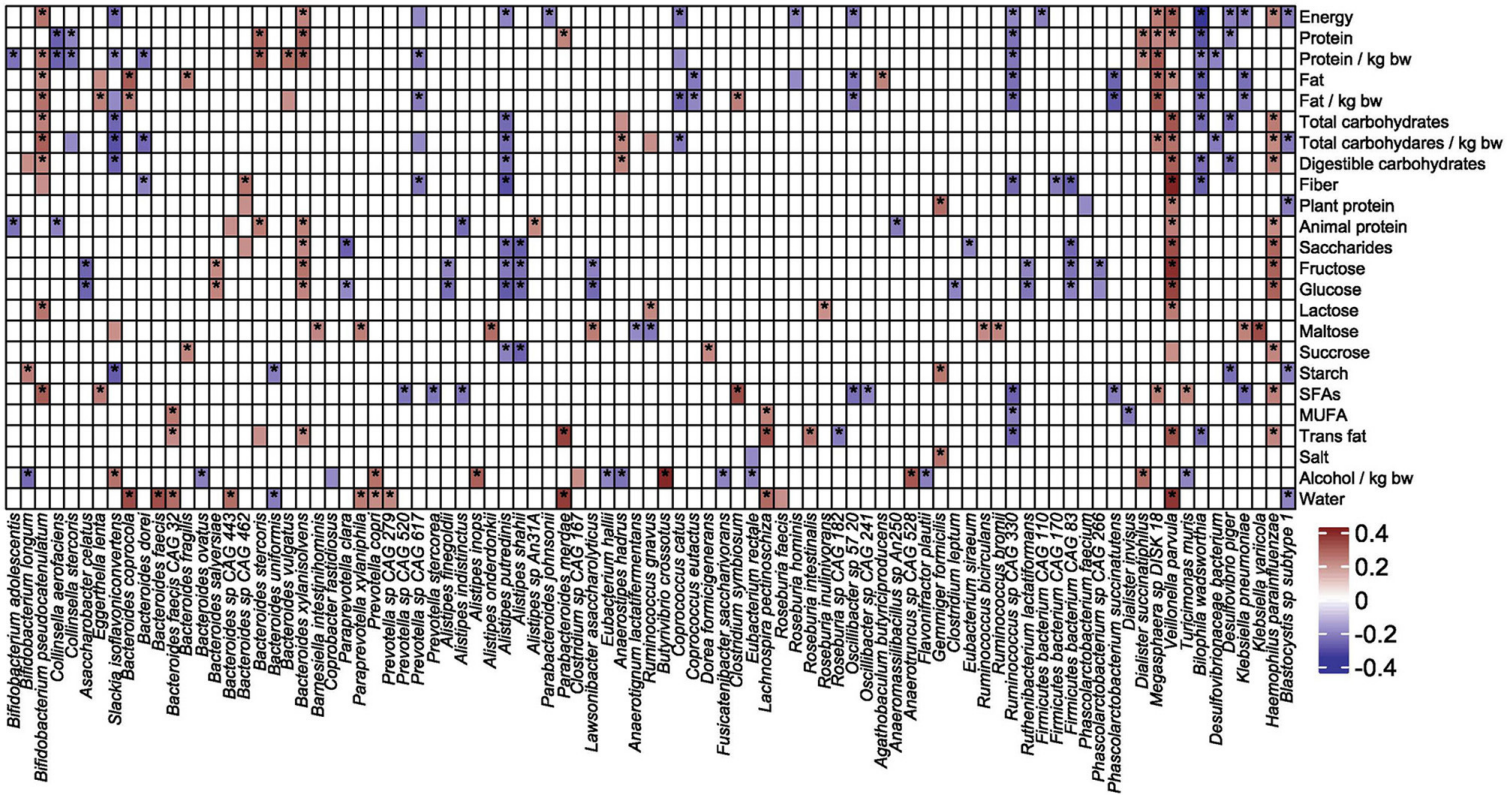
Spearman's correlation coefficients showing correlations between macro dietary measures and species abundance. Values with corresponding *p*-value lower than 0.05 are denoted with asterisk.

Two hundred fifty-three macronutrient-species pairs were showing Spearman's rank correlation coefficients between 0.2 and 0.39. Of these, at least one of the 25 dietary measures is associated with the abundance of at least one of the 91 bacterial species (Figure 10). Nine species were associated with seven or more nutrients. *Veillonella parvula, Haemophilus patainfluenzae, Bifidobacterium pseudocatenulatum*, and *Bacteroides xylanisolvens* correlated positively with 16, 11, 10, and eight nutrients, respectively, and *Desulfovibrio naceaebacterium, Alistipes putredinis, Ruminococcus sp CAG330*, and *Bilophila muris* correlated negatively with nine different nutrients. These findings confirm the key role of diet in the functional composition of the gut microbiota.

The correlation between species abundance and nutrients was also assessed using LINDA. As shown in Table 4, nine species (*Bacteroides coprocola, Bacteroides faecis, Bacteroides sp CAG443, Bifidobacterium pseudocatenulatum, Lachnospira pectinoschiza, Parabacteroides merdae, Paraprevotella xylaniphila, Prevotella copri*, and *Veillonella parvula*) were correlated with eight nutrient measures, whereas seven species correlated with the amount of drinking water. All associations were positive.

**Table 4:** Bacterial species associated with nutrient parameters, as signified by the LINDA model.

**Species**	**Nutrient**	**log2-FC**
*Bacteroides coprocola*	Fat	0.85
*Veillonella parvula*	Total carbohydrates	1.03
*Bifidobacterium pseudocatenulatum*	Total carbohydrates g/kg bw	1.45
*Veillonella parvula*	Fructose	1.14
*Veillonella parvula*	Glucose	1.15
*Bifidobacterium pseudocatenulatum*	Lactose	1.40
*Bacteroides sp CAG443*	Alcohol g/kg bw	0.72
*Prevotella copri*	Alcohol g/kg bw	1.82
*Veillonella parvula*	Water	1.172
*Bacteroides coprocola*	Water	0.80
*Parabacteroides merdae*	Water	1.24
*Lachnospira pectinoschiza*	Water	1.18
*Bacteroides faecis*	Water	0.91
*Paraprevotella xylaniphila*	Water	0.87
*Bacteroides sp CAG443*	Water	0.67

## 4. Discussion

Both host genetics and environmental factors modulate the structure and function of the gut microbial community. Of the latter, exercise and diet affect the gut microbiota of professional athletes and highly fit individuals, who show more dynamic changes compared with their less fit or sedentary counterparts (43). For the first time, our study presents an integrated view of the relationship between physical activity levels and dietary habits of esports players and the composition and function of their gut microbiome. The study included two reference groups: healthy university physical education students and professional athletes.

Although esports players had lower muscle mass and higher fat mass than physical education students, which might suggest a more sedentary lifestyle, neither the α- nor the β-diversity of the gut microbiota differentiated these two groups. Instead, β-diversity significantly differentiated professional athletes from both of these groups, and α-diversity distinguished professional athletes from esports players. Similarly, the α-diversity of professional rugby players was statistically higher than that of high-BMI, but not low-BMI, controls (44). In female endurance runners (45) and healthy men aged 60–70 years with lifelong experience of strenuous endurance training (46), the β-diversity, but not the α-variety, was affected.

*Bacteroides*-driven enterotype 1 is typical for a “Western” type diet enriched in protein and animal fat, whereas *Alistipes*-driven enterotype 2 is associated with long-term consumption of carbohydrate-rich diets (8). Furthermore, while the bacterial cluster of enterotype 1 acquires energy primarily from carbohydrates *via* the glycolysis and pentose phosphate pathways, bacteria clusters of enterotype 2 and 3 may also degrade mucin glycoproteins of the gut mucosal layer (7). The three dominant enterotype clusters were identical in the esports player and student groups, but enterotype 1 was dominant; however, enterotype 1 was under-represented and enterotype 2 was over-represented in male participants with medium to high levels of physical activity compared with those with low levels. Consistent with this, enterotype 1 was not found in the athlete group. By contrast, previous studies show that neither continuous physical activity performed at low doses by premenopausal women (12), nor extensive exercise training conducted by female endurance runners (45), modified the enterotype clusters. In addition, while the B/F ratio did not differentiate esports players from students, professional athletes had a significantly lower B/F ratio than both of these groups. This confirms our earlier findings showing that Polish athletes have a higher abundance of Firmicutes than controls (33). In healthy young adults, the B/F ratio correlates significantly with cardiorespiratory fitness, a powerful indicator of athletic performance and health, but not with other fitness parameters, nutritional intake, or anthropometric variables (43). Also, daily exercise enriches Firmicutes in the gut of young children and adolescents (47). However, some studies report a decreased B/F ratio in obese individuals, although other studies showed no changes, or even an increased B/F ratio, in obese animals and humans (47). Further studies are needed to assess whether an altered B/F ratio can be used as an exercise signature.

On the species level, the abundance of nine bacteria differentiated esports players from students; all were under-represented in esports players, while as many as 31 and 45 bacteria differentiated professional athletes from students and esports players, respectively. However, only *Parabacteroides distasonis* species differentiated all three experimental groups from each other, with the lowest abundance in esports players and the highest in controls. This species contributes indirectly to methane, acetic acid, and anti-inflammatory succinic acid production (48), thereby playing a protective role against colon cancer development and human obesity (49). Its contribution to autoinflammatory diseases remains ambiguous (50).

A higher abundance of *Akkermansia* species, which have potentially beneficial effects, were found in athletes when compared with the two other groups. *Akkermansia* is associated with overall good health and is less abundant among obese individuals (51). Additionally, *A. muciniphila* increases gut barrier function (52), promotes the release of human glucagon-like peptide-1 (GLP-1), downregulates the production of pro-inflammatory cytokines, and controls fat storage (53, 54). The following abundant species, *Roseburia hominis*, is a known producer of butyrate (55). In turn, *Anaeromassilibacillus sp An250* is associated with both low BMI (56) and high-intensity physical activity (57). Lactate, a by-product of skeletal muscle energy metabolism during exercise, is transported to the gut, where it enhances the growth of propionate-producing bacteria, including *Veillonella* (58). Here, we found that the *Veillonella* genus correlated positively with muscle mass and fiber and carbohydrate consumption. Additionally, two potentially harmful bacteria, *Bilophila wadsworthia* and *Ruminococcus gnavus*, were under-represented in professional athletes. *Bilophila wadsworthia* is a bile-tolerant and sulfite-reducing bacteria associated with a high-fat animal-based diet. Hydrogen sulfide produced by *B. wadsworthia* is a well-known pro-inflammatory factor (59, 60). *Ruminococcus gnavus* is connected to Crohn's disease through its pro-inflammatory product, glucorhamnan polysaccharide (61), and is associated with metabolic syndrome (62). By contrast, the microbiome of esports players and students was enriched in beneficial *Bacteroides* species: *B. thetaiotaomicron* contributes to an increase in mucus production and strengthening of the epithelial barrier (63), and has anti-inflammatory properties that inhibit the development of Crohn's disease (64), while *B. uniformis* is negatively correlated with physical activity in older adults (65), alleviates obesity in conjunction with an appropriate diet (66) and enhances endurance exercise performance (67). To summarize, our findings confirm changes in the relative abundance of different taxa reported in professional rugby players (44), marathon runners (58), professional cyclists (68), and other sports persons (69), as well as in elderly healthy individuals with a lifelong history of endurance training (46) and premenopausal women after continuous physical activity (12).

The metabolic activity of the gut microbiota results from the degradation of dietary carbohydrates, lipids, and proteins that exert both local and systemic activity; these include SCFAs and branched-chain fatty acids, alcohols, ammonia, amines, sulfur compounds, phenols and indoles, glycerol, and choline derivatives (3). SCFAs, like butyrate, acetate, and propionate, are the most abundant fecal metabolites. Butyrate serves as an energy source for colonocytes, improves the integrity of intestinal epithelial cells, increases mucin and cytokine production, and induces differentiation of naive T cells. Acetate and propionate also aid in anti-inflammatory processes and cytokine production. In addition, after adsorption from the gut lumen, SCFAs modulate metabolic responses at different sites, including skeletal muscle (70), and their excess can be incorporated into the gluconeogenic and lipogenic processes (71). As SCFAs play a pivotal role in whole-body energy metabolism, and their levels correlate with variations in the gut microbiota (7), it is not surprising that high levels of physical activity enrich taxa known to participate in SCFA production (70). However, although a 6-week exercise training program in lean sedentary people enhanced fecal SCFA concentrations (13), and a literature search confirmed increased production of gut SCFAs in response to exercise (51), none of the SCFAs differentiated our studied groups. Instead, five SCFAs differentiated between enterotypes. Of these, propanoic, isobutyric, pentanoic, and hexanoic acid differentiated between *Alistipes-* and *Bacteroides*-driven enterotypes, acetic and propanoic acid differentiated between *Prevotella-* and *Alistipes-*driven enterotypes, and pentanoic acid differentiated between *Prevotella-* and *Bacteroides*-driven enterotypes.

Increased bacterial metabolic processes in the distal parts of the colon may also result from higher availability of amino acids (72). In contrast to fecal SCFAs, the amounts of each of the nine amino acids studied herein differentiated esports players from students, whereas the amounts of four amino acids differentiated professional athletes from students, while methionine differentiated esports players from the two other groups. However, the role of interrelationships between gut amino acid levels and the structure and function of the gut microbiome has not been clarified.

On the functional level, we found no differences between esports players and students, although several metabolic pathways were over-represented in professional athletes compared with esports players and students; these included *Fermentation, Amino acid Biosynthesis and Degradation, Carbohydrate Biosynthesis and Degradation, Fatty Acid Biosynthesis and Degradation*, and the *TCA Cycle* pathways. The other pathways, including *Cell Structure Biosynthesis, Nucleoside and Nucleotide Biosynthesis*, and *Generation of Precursor Metabolites and Energy*, appears to differentiate between enterotypes. The results of our functional analysis correspond with other findings. As demonstrated previously, the diverse responses of glycemic control and insulin sensitivity to training results from shifts in microbial fermentation (73). Similarly, high-intensity interval training enhances amino acid biosynthesis pathways (74). Likewise, the microbiomes of rugby players are enriched for carbohydrate metabolism, amino acid biosynthesis, and SCFAs synthesis pathways (44). SCFAs, as precursors for lipid or carbohydrate synthesis, enter the TCA and are used as substrates for lipogenesis or gluconeogenesis, respectively (70). Therefore, the bioavailability of gut SCFAs is considered to be a potential regulator of skeletal muscle metabolism. Peak oxygen uptake during cardiorespiratory fitness exercise correlates with changes in gut microbiota diversity. Diversity is associated with microbial metabolic functions, including fatty acid biosynthesis (75). Additionally, the microbiome of athletes showed a significant increase in the capacity for nucleotide biosynthesis and improved cell structure, which play a role in energy demands essential in endurance sports (44).

### 4.1. Conclusion

Overall, although our findings are consistent with those of previous reports in many respects, the differences between esports players and physical education students concerning lifestyle and dietary habits appear to have little effect on most gut microbiota parameters. Nevertheless, differences in the abundance of several bacterial species and marked differences in fecal amino acid concentrations between these two groups were noted. However, the main limitation of this study, as well as other similar studies, relates to the self-reporting of physical activity time and dietary habits. People tend to overestimate their activity levels (76) and underestimate their food intake. Therefore, future studies should be conducted based on strictly controlled dietary intake and physical activity levels to fully determine their effect on gut microbiota composition.

## Data availability statement

The data presented in the study are deposited in the SRA at http://www.ncbi.nlm.nih.gov/bioproject/885289, repository, accession number PRJNA885289.

## Ethics statement

The studies involving human participants were reviewed and approved by Institute of Oncology Local Bioethics Board. The patients/participants provided their written informed consent to participate in this study.

## Author contributions

MK: data curation, formal analysis, visualization, writing–original draft, and writing–review and editing. BF: conceptualization, methodology, resources, validation, writing–original draft, and writing–review and editing. AB, PC, KB, MG, AK, and MP: investigation. NZ-L: investigation, visualization, and writing–review and editing. BZ and MSk: resources. MSz: resources and writing–review and editing. MM: visualization and writing–review and editing. JO: conceptualization, funding acquisition, project administration, resources, writing–original draft, and writing–review and editing. All authors contributed to the article and approved the submitted version.

## Supplementary material

Supplementary Figure 1 Between-class analysis visualization, including representation of cluster drivers **(C)** and individual samples within clusters **(B)**, as well as the choice of optimal cluster number **(A)**.

Supplementary Table 1 Gaming experience of esports players.

Supplementary Table 2 Characteristics of esports players and control groups.

Supplementary Table 3 Energy and nutritional value of the diets of esports players and students.

Supplementary Table 4 Network parameters, calculated by NetConfer, for three enterotypes: *Prevotella* (P)-, *Alistipes* (A)-, and *Bacteroides* (B)-driven.

Supplementary Table 5 Differences between esportsmen and healthy students, and professional sportsman, calculated by the LINDA model.

Supplementary Table 6 Kruskal-Wallis test *p*-values for comparison of short chain fatty acid levels in students and esports playares, presented as the median and IQR (in parentheses).

Supplementary Table 7 Functional differences between professional sportsmen, esportsmen, and healthy control students, as calculated by the LINDA model.
