# Supplementary figures and images for: Characteristics of the gut microbiome in esports players compared with those in physical education students and professional athletes
Source: Front Nutr. 2023 Jan 16;9:1092846. doi: 10.3389/fnut.2022.1092846 (PMC9884692; doi:10.3389/fnut.2022.1092846)

a

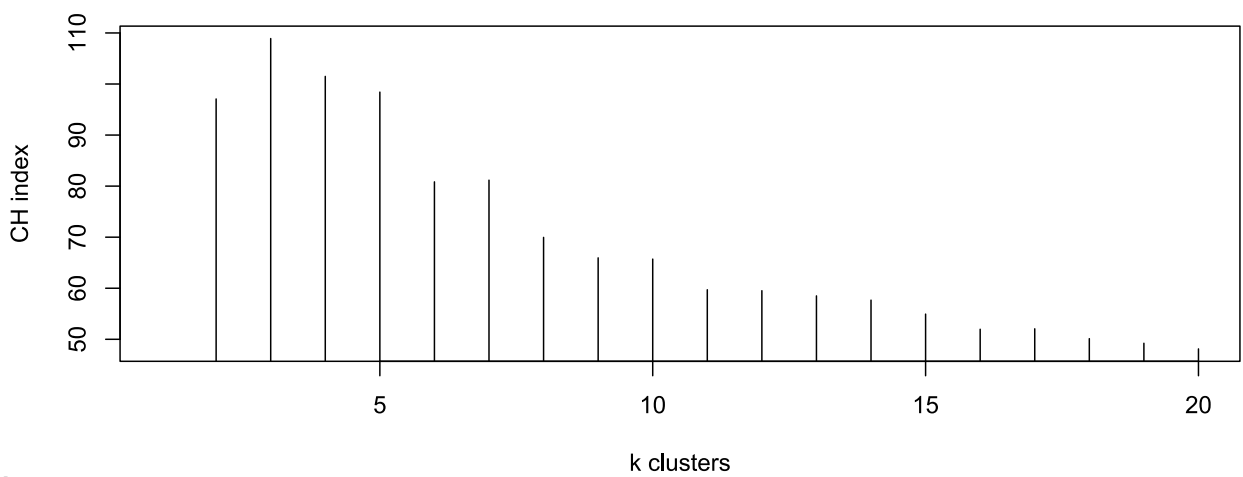

b

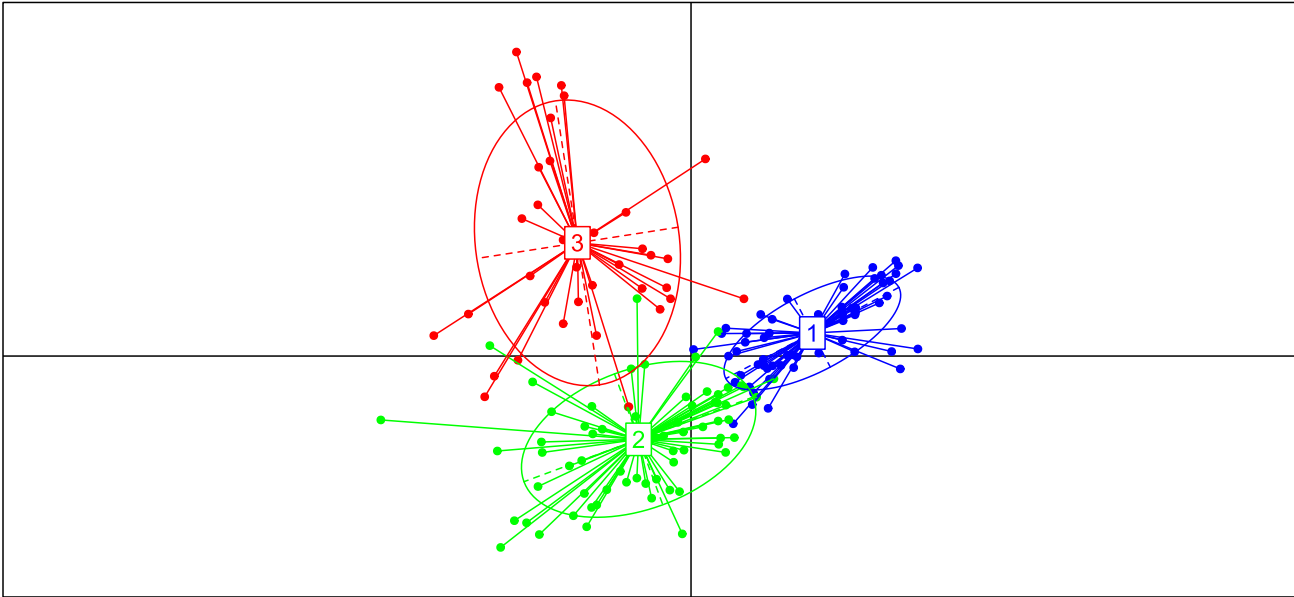

c

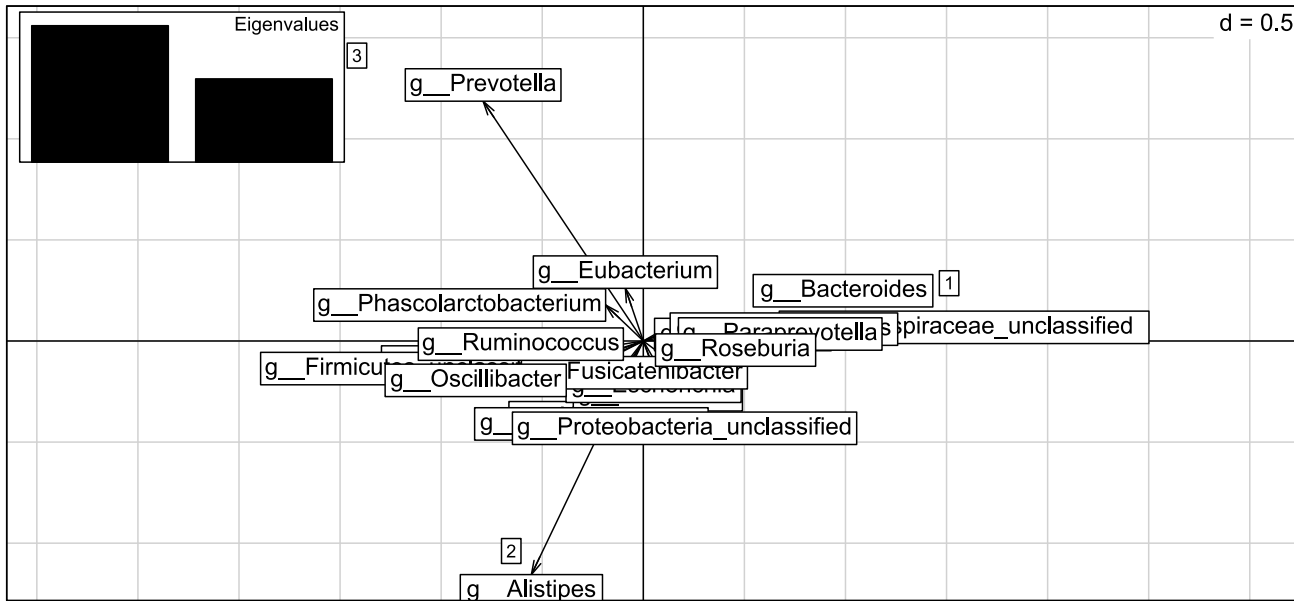

Supplement: Supplementary Figure 1 — Between-class analysis visualization, including representation of cluster drivers (C) and individual samples within clusters (B), as well as the choice of optimal cluster number (A). [file Image_1.PDF]
